# Waveguide Spectroscopy for Differentiation of Bacteria

**DOI:** 10.1021/acs.analchem.5c02661

**Published:** 2025-09-26

**Authors:** Arvid Angelsten, Pontus Forsberg, Håkan Engqvist, Wei Xia, Mikael Karlsson

**Affiliations:** Department of Materials Science and Engineering, 8097 Uppsala University, 751 03 Uppsala, Sweden

## Abstract

Mid-infrared spectroscopy allows precise and label-free molecular fingerprinting of the chemical composition of bacteria. Waveguide spectroscopy promises several benefits compared to conventional ATR-FTIR spectroscopy, such as higher sensitivity and the potential for miniaturization. In this study we demonstrate mid-infrared spectroscopy of lipopolysaccharides and intact bacteria cells on a diamond waveguide with quantum cascade lasers as light source. The diamond waveguide with aluminum nitride as undercladding was tuned with a thin silicon film to specifically target the polysaccharide part of the mid-infrared spectrum (1200–1050 cm^–1^). It was shown to have good performance when measuring in aqueous solutions, with an order of magnitude sensitivity enhancement compared to conventional ATR-FTIR equipment. Due to the relatively low water absorptions and large differences in the absorptions of bacterial lipopolysaccharides in this spectral region, it was possible to differentiate between lipopolysaccharides obtained from different strains of *Escherichia coli*, as well as between Gram-positive and Gram-negative bacteria.

## Graphical abstract

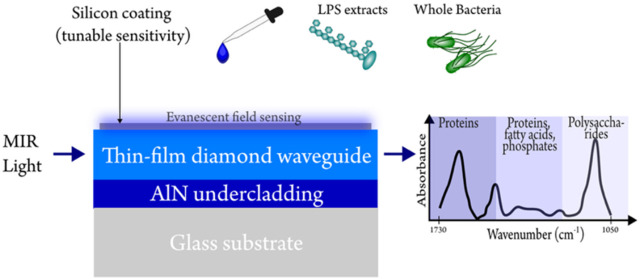

## Introduction

Vibrational spectroscopy, including infrared (IR) and Raman spectroscopy, is widely used for studying biological samples. When studying bacteria, the benefits of these techniques include simple sample preparation and the possibility to obtain chemical fingerprints of the cellular composition without requiring labels or dyes.^1^ The applications range from bacteria detection, genus and strain typing to antimicrobial susceptibility testing and drug-cell interactions.^2–4^ Raman spectroscopy has been demonstrated for detection of bacteria in various aqueous-based media, such as urine, drugs and milk.^5–7^ In contrast, IR spectroscopy of aqueous samples is more challenging because of the strong water absorptions around 3500 and 1640 cm^–1^, due to OH stretching and bending vibrations.^8^ IR spectroscopy provides complementary information to Raman spectroscopy and there is growing interest in advancing IR technology for biological studies in water environments.

IR spectroscopy holds promise as a rapid and noninvasive technique for bacterial typing and antimicrobial testing, enabling faster clinical decision-making. The literature reports a multitude of studies demonstrating the possibility to correlate mid-infrared (MIR, 4000–400 cm^–1^) absorption patterns to differences of molecular composition of bacteria. IR spectroscopy of bacteria is typically performed with Fourier Transform Infrared (FTIR) spectroscopy either in transmission mode or using attenuated total reflectance (ATR).^9–11^ To improve the reproducibility the bacteria are usually fixed on transmission slides or the ATR accessory by drying prior to analysis. However, dehydration of biological samples leads to changes in the molecular structure.^12^ MIR spectra of bacteria are complex, containing overlaid spectral signatures from various biomolecules such as fatty acids, proteins, nucleic acids and polysaccharides. This complexity can hinder qualitative spectral interpretations, so focusing on a spectral region with clear signature spectra is important. Machine learning and AI have enabled correlations between MIR absorption patterns and bacterial genotype or antimicrobial resistance. In these studies, the subregion 1200–900 cm^–1^ have repeatedly been shown to contain the most useful spectral features,^13–21^ primarily due to polysaccharide vibrations.^22^ The low water absorption at these wavenumbers have further been shown to improve the typing accuracy compared to other spectral regions.^23^

Bacteria are composed of a cytoplasm and a cell envelope consisting of the cytoplasmic membrane and the peptidoglycan-rich cell wall. At the highest level, bacteria can be classified as either Gram-negative or Gram-positive, depending on the general composition of their cell wall. Gram-positive bacteria have a thick peptidoglycan layer that constitutes around 60–90% of the cell wall’s dry weight, consisting mainly of polysaccharides, peptides and teichoic acids.^24^ Gram-negative bacteria have a much thinner peptidoglycan layer, constituting around 10–20% of the cell wall’s dry weight, and lack teichoic acids, but instead have an outer membrane containing lipopolysaccharides (LPS). Peptidoglycan and LPS contain polysaccharides, and are mainly located in the bacterial cell envelope. They are important for distinguishing between bacteria taxonomically and at the serological level, or to study their antimicrobial resistance properties. LPS are essential components of the outer membrane of Gram-negative bacteria, with critical roles in maintaining structural integrity and protecting bacteria from external stresses.^25,26^ The structure of LPS molecules vary considerably between bacteria strains. LPS have three main parts: a lipid anchor with fatty acids and a disaccharide backbone, a core polysaccharide, and an O-antigen made up of repeating oligosaccharide units. The LPS structure is known to correlate with ability of the bacteria to prevent binding of proteins, to evade detection by the immune system, and acts as an additional permeability barrier for antibiotics.^26^ LPS is a potent endotoxin triggering strong immune responses in humans, playing important roles in sepsis and infections. Detection of LPS in blood can be used as an indicator of an ongoing infection and to monitor sepsis, and analysis of its structure provides important information for developing new diagnostics tools and antibiotics. Pharmaceuticals are regularly tested for LPS to avoid contamination.^27^ Detection is usually done by Limulus Amebocyte Lysate based assays or immunoassays such as ELISA,^28,29^ with a limit of detection (LOD) on the order of nano- or even picograms/ml.^29^ These methods typically do not differentiate between different LPS or require a tailored assay for each specific LPS. MIR spectroscopy on its own is unlikely to reach such low detection limits, but the wealth of data in the spectrum makes it a valuable complementary measurement for identification rather than detection.

Directly measuring live bacteria cells or LPS in aqueous environments holds several benefits compared to studying dried samples. In aqueous solutions, the native conformation and function of the cells are preserved, providing MIR information that is biologically relevant and accurate. This approach offers the possibility of real-time monitoring and quicker diagnostics. Despite the strong water signature in the MIR spectrum, analysis of water solutions is feasible by using light sources with high brightness like synchrotron radiation,^30^ or by reducing the optical path length in the sample by e.g., pressing the sample in between transmission windows or by ATR. In ATR-FTIR, total internal reflection of the light in the ATR crystal produces an exponentially decaying evanescent field that extends into the sample at the crystal interface, with typical penetration depths on the order of micrometers. By reducing the interaction with the analyte to the evanescent field, ATR allows the study of highly absorbing samples, such as aqueous solutions while keeping sample preparation simple. Depending on the absorbance of the sample, the interaction strength (the effective path length in the sample) can be optimized by varying the incidence angle or the number of internal reflections, to maximize the signal-to-noise ratio (SNR). Accessories for large numbers of reflections however typically require larger sample volumes. ATR-FTIR has been used to study biofilm formation in liquid cultures, to detect bacteria in drinking water, and monitor microbial chemistry variations during bacterial growth.^31–33^

Recent studies highlight the potential of thin-film wave-guides for absorption spectroscopy of biological samples in the MIR using Quantum Cascade Lasers (QCLs) as a light source.^34,35^ It offers potential advantages over traditional ATR-FTIR instruments in terms of sensitivity and SNR. In contrast to ATR-FTIR, where the evanescent field is only present at the locations of the discrete number of internal reflections, the evanescent field of a thin waveguide spreads out along the waveguide surface increasing the light-analyte interactions, allowing increased sensitivity per unit length. Modern QCL systems offer high resolutions spanning most of the MIR, high brightness, and can be miniaturized and potentially integrated on a chip. MIR optoelectronic technology is relatively immature compared to corresponding technologies in the visible and near-infrared regions, and the potential of miniaturized sensors in the MIR has not yet been realized. MIR waveguide spectroscopy has previously been demonstrated for measuring proteins in phosphate buffered solution^36^ and caffeine in espresso.^37^ More recently it has been used to study biofilm formation of *Escherichia coli* (*E. coli*) on diamond and gallium arsenide waveguides.^38^

In this study, we characterize LPS extracts and intact bacteria cells in water solutions using MIR absorption spectroscopy on a thin-film waveguide platform. To this end, we employ a novel thin-film diamond waveguide combining the low sensitivity required to measure at the water absorption around 1640 cm^–1^ (which overlaps the amide I band important for analyzing proteins) with high sensitivity in the polysaccharide region of the MIR spectrum. In previous works it has been established that this region contains the most valuable MIR information correlating to bacterial structural differences and antimicrobial resistance properties. We demonstrate here for the first time the use of waveguide spectroscopy for differentiation of bacterial extracts and whole bacteria using the polysaccharide MIR fingerprint.

## Methods

### Design and Simulation of Waveguide

The waveguide for this study was specifically designed to be sensitive in the wavenumber range of 1200–1050 cm^–1^. Previous diamond waveguides used for MIR spectroscopy have had difficulty to access this range while maintaining high sensitivity, due to losses in the undercladding. By sensitivity we mean the absorption loss to the analyte for a given absorption coefficient of the analyte. In the case of silicon dioxide (SiO_2_) undercladding,^37–39^ absorption losses become too high in this range, and around 1050 cm^–1^ waveguiding is not possible even in principle, since the refractive index of SiO_2_ exceeds that of diamond. While making a waveguide that is not supported on an undercladding material is possible^40^ such waveguides are fragile and not suited for the repeated cleaning by mechanical wiping required to remove biological samples. To reduce absorption in the undercladding, we instead used aluminum nitride (AlN) undercladding^41^ which has an extinction coefficient ∼100 times lower than SiO_2_ at 1100 cm^–1^. AlN still has some absorption in this range, and in our previous work with a 5 μm thick diamond waveguide, measurements in water were not possible below 1150 cm^–1^. To improve the usability and the stability of the sensor and to increase the signal strength, we here use a thick diamond waveguide (19 μm), which makes it significantly easier to couple light into the waveguide and also reduces interaction with the undercladding. There is a risk of instability when using multimode waveguides, due to small variations in the input changing the coupling into the different modes. We have however had less stability and repeatability issues when coupling into the lower modes of a thick waveguide than when using a thin single mode waveguide where small changes in the input beam will cause fluctuations in how much light is coupled into the waveguide. Unfortunately, interaction with the sample is reduced when using the lower modes of a thick waveguide, so the sensitivity is much lower. The sensitivity is affected by the proportion of the guided light that is present in the evanescent field and also the length of the waveguide. Sensitivity can be increased by coupling light into higher waveguide modes or increasing the waveguide length, but this again increases losses in the undercladding. By sputtering a thin film of silicon (Si) on the diamond surface we could increase the sensitivity in the specific wavenumber range of interest while keeping losses in the undercladding low.^39^ The higher refractive index film moves the electromagnetic field in the waveguide closer to the surface and increases the power in the evanescent field. A schematic view of the waveguide geometry can be seen in Figure 1. Silicon is somewhat absorbing in the wavelength region of interest, but the thin film and relatively short waveguide keeps the absorption manageable. The sensitivity of the transverse-electric (TE) modes can be enhanced by a thinner film than the transverse-magnetic (TM) modes. Since a thinner film is easier and cheaper to deposit and also gives lower losses, we designed our waveguide for the TE modes. To get good coupling into the diamond waveguide, the edges and a narrow border around the top of the waveguide were not coated with Si.

**Figure 1:**
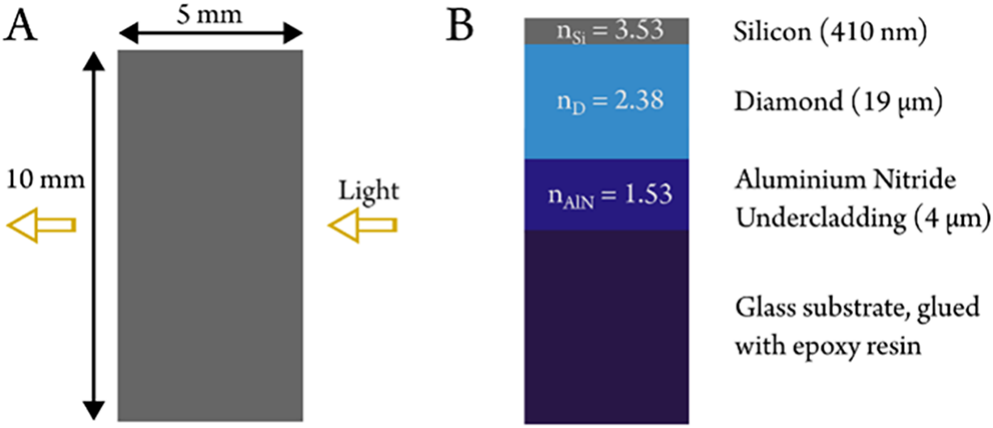
Waveguide dimensions in (A) the lateral plane and (B) the vertical cross section (film thickness not to scale). The refractive indices at 1100 cm^–1^ are also included for the optically active layers.

To determine the optimal thickness of the silicon film, we first used a simple finite difference calculation to find the mode profiles of the waveguide (in water) with a range of film thicknesses. We then used finite difference time domain field propagation with the simulation package MEEP,^42^ to determine which modes were likely to be excited in our experiment. This is described in more detail in the online Supporting Information. To estimate the sensitivity for a given length of waveguide, the proportion of mean power flux in the analyte, compared with the total power flux in the waveguide, was calculated. This is proportional to the effective thickness in ATR spectroscopy^43^ per unit length of waveguide. (For a waveguide the absorbance is proportional to the length of the waveguide in contact with the sample rather than the number of reflections as in ATR.) The simulations showed that a film thickness around 400 nm should give peak sensitivity in the region of interest. The proportion of power flux in the analyte with a 410 nm thick Si film (to match the fabricated waveguide below) is shown in Figure 2. It is above 2% for the whole range 1200–1050 cm^–1^ with a peak of 3.3% at 1087 cm^–1^. Without a silicon film, only ∼0.05% of the power would be in the evanescent field. So, compared to a waveguide with no Si film the sensitivity is 65 times higher at 1087 cm^–1^. Between 1600 and 1700 cm^–1^, our waveguide is only around twice as sensitive as previous diamond waveguides with a similar thickness and without a Si film.^37,38^

**Figure 2:**
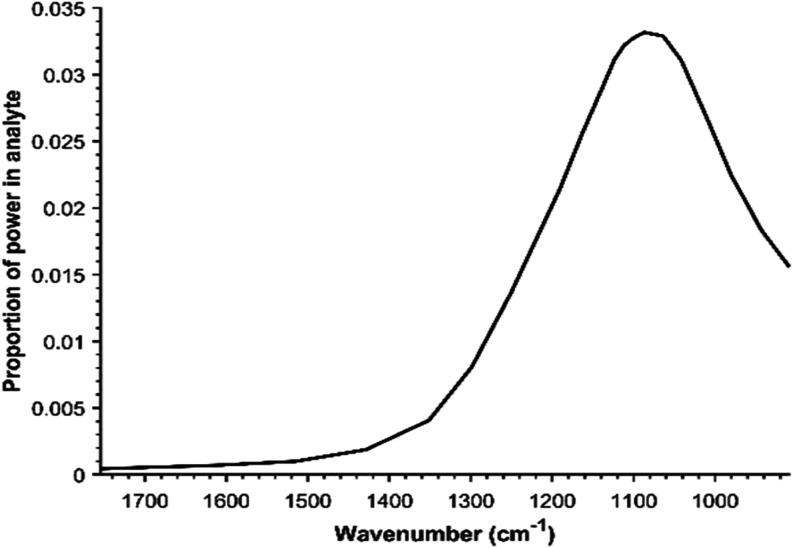
Simulated sensitivity spectrum of the diamond waveguide with a 410 nm silicon film. Proportion of power flux in the evanescent field in the analyte.

### Waveguide Fabrication

The waveguide fabrication process was similar to a combination of those presented in two previous publications.^39,41^ A 19 μm thick diamond film was deposited through chemical vapor deposition on a silicon wafer and polished to less than 10 nm roughness (Ra) by Diamond Materials GmbH. The wafer was laser cut to produce smaller samples and a 5 mm × 10 mm piece was used for the waveguide presented here. The sample was cleaned by wiping and rinsing with isopropyl alcohol followed by 10 min in piranha solution (a mixture of sulfuric acid and hydrogen peroxide). AlN was deposited on the sample by reactive sputtering in a Von Ardenne magnetron sputter system with 900 W pulsed DC power at 2 μBar pressure with gas flows of 10 sccm argon and 30 sccm nitrogen. Deposition was done in three steps (30, 30, and 26 min) and the sample and sputter system were allowed to cool to room temperature in between. The thickness of the AlN film was measured to 4.0 μm. A piece of 1 mm thick glass was cut to the same dimensions as the sample and glued to the AlN using epoxy resin (Biltema Express-epoxy, cured at room temperature). The longer edges of the sample were then mechanically polished on a diamond coated polishing wheel (Nova Diamant AB). After polishing, the silicon substrate that the diamond was grown on was removed by dry etching in a sulfur hexafluoride and argon plasma (PlasmaTherm SLR Inductively Coupled Plasma system). The silicon film was deposited on the waveguide by sputtering in the same magnetron sputter system that was used for AlN deposition (500 W pulsed DC power in 1.5 μBar argon). A 3D printed frame was used to mask the sides and a couple of hundred μm around the edges of the waveguide during sputtering. The film thickness was measured with a stylus profilometer on a piece of silicon masked with Kapton tape that was sputtered together with the waveguide and the thickness was 410 ± 10 nm. The final waveguide is ∼4.9 mm long with ∼4.5 mm coated with Si.

### Waveguide Spectroscopy Setup

The spectroscopy setup is shown in Figure 3. A QCL system (MIRcat, Daylight Solutions) was used as light source with three optically coupled QCL chips covering the regions (i) 1730–1425 cm^–1^, (ii) 1450–1125 cm^–1^ and (iii) 1275–1050 cm^–1^. In order to only excite TE modes in the waveguide a polarizer (Edmund Optics, BaF_2_ holographic wire grid) was placed in the beam path before the waveguide. Three zinc-selenide lenses (Thorlabs) were used to couple light into the waveguide (*f* = 12.7 mm) and to collect the output light (*f* = 12.7 mm and *f* = 25.4 mm) onto a mercury cadmium telluride (MCT) detector (VIGO System PVMI-4TE-12). The detector signal from the detector and trigger signals from the laser were collected with an oscilloscope (National Instruments PXIe-5114).

**Figure 3:**
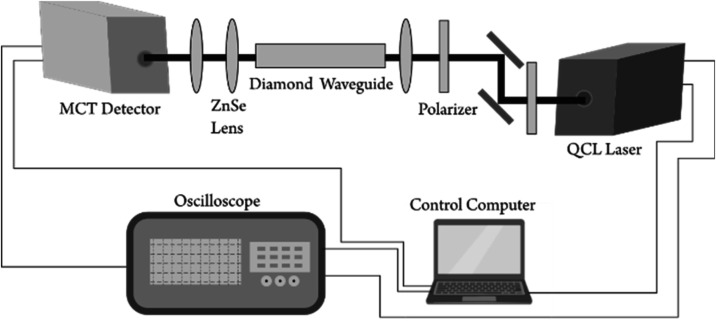
Experimental setup.

### Sample Preparation

#### Lipopolysaccharide Extracts

Phenol-phase extracted lipopolysaccharides from the *E. coli* strains O111:B4 and O26:B6 were purchased from Sigma-Aldrich. The extracts were dissolved in distilled (DI) water to a concentration of 5 mg/mL. Lower concentration samples (1–4 mg/mL) were obtained by dilution.

#### Cell Cultures

Intact cells from the nonpathogenic bacteria *E. coli* MG1655 and *Staphylococcus aureus* ATCC 33591 (*S. aureus*) were used in this study. *E. coli* is Gram-negative while *S. aureus* is a Gram-positive bacterium and hence lacks an outer membrane. The bacteria were streaked on Lysogeny broth agar plates, and grown overnight at 37 degrees. Bacterial mass (roughly 100 μg) was harvested with inoculating loops and placed in DI water. The cells were washed by centrifugation at 4000 rpm for 4 min, removing the supernatant and resuspending the cells in DI water. This washing procedure was repeated three times. After the last centrifugation, the final bacterial solution was obtained by adding 50 μL DI water to the cell pellet. Prior to measurements the solutions were vortexed to ensure homogeneity.

### Data Acquisition and Processing

Spectra were collected using a custom LabView script synchronizing the QCL output and detector response. The laser was operated in pulsed mode with pulse frequency of 100 kHz. The output signal was obtained as voltage readings on the oscilloscope sampled at 125 MHz and calculated as the difference between the mean voltages during and between the pulses. More details about the data collection can be found in ref 41.

#### Sample Measurements

Fifteen μL of the sample solution was pipetted onto the waveguide, covering the whole length, and measured with 0.5 cm^–1^ resolution and 3–10 scans for each QCL chip (3 scans for the isopropyl alcohol measurements, 5 scans for the LPS measurements and 10 scans for the bacteria measurements; total measurement time was 45–150 s with our QCL). Between each set of measurements, the waveguide was thoroughly wiped clean using 70% isopropyl alcohol and distilled water. For measurements of the LPS solutions, we let the water evaporate and measured every 10 min during a 30 min period, in order to see the influence of increased concentrations. DI water was used as the background for all measurements. For each bacterial sample, the measurement was repeated several times and the waveguide was cleaned between measurements. The measured spectra were screened and those that showed obvious errors (typically due to outside disturbance during the measurement, such as waveguide movement when cleaning between background and sample) were discarded.

#### Baseline Correction

To minimize baseline drifts between sample and background measurements, we performed baseline correction using the Asymmetric Least Squares smoothing (ALS) algorithm.^44^ The ALS algorithm calculates the baseline by solving a penalized least-squares problem, where the weights are asymmetrically assigned to the residuals to make the baseline follow the lower envelope of the data. The relevant parameters are the smoothing parameter, λ, and the asymmetry parameter, p, that controls the emphasis given to negative residuals. The smoothing and asymmetry parameters were optimized manually to ensure accurate subtraction without distortion of spectral features. Optimal parameters were p = 10^–2^ and λ = 10^5^–10^7^.

#### Smoothing and Noise Reduction

The spectra were further processed to reduce high-frequency noise in the signal. Fast-Fourier transform (FFT) filters were applied to the spectra using the python libraries Numpy and Scipy.^45,46^ Since the noise levels vary between spectral regions, due to different water and atmospheric absorptions (mainly CO_2_ and water vapor in the path of the laser beam), the optimal FFT parameters had to be adjusted for different QCL chips and between measurements.

#### Normalization and Absorbance Calculation

Each sample spectrum was normalized against a background spectrum obtained for DI water in similar conditions. The absorbance was calculated according the formula

$$A\left(f\right)=-\log_{10}\;\frac{I\left(f\right)}{I_{0}\left(f\right)}$$

where *f* is the frequency, *I is the sample signal* and *I*_0_ is the background signal.

#### Quantitative Analysis

For quantitative analysis and to estimate the LOD, the LPS solutions were measured with different concentrations and the absorption at a characteristic peak (e.g., 1080 cm^–1^) was plotted against concentration. A straight line was fitted to the data, and the fitting parameters were used to estimate the LOD according to

$$\mathrm{LOD}=3.3\frac{\sigma }{S}$$

where σ is the standard deviation at the *y* intercept of the fitted line, and *S* is the slope.

#### Principal Component Analysis

Principal component analysis (PCA) was computed on the set of waveguide spectra of the different bacteria between 1250 and 1070 cm^–1^. Prior to analysis the spectra were standardized. PCA was carried out in Python using the module scikit-learn.^47^

### Comparison with ATR-FTIR

For comparison, the same sample solutions analyzed with the waveguide were measured with single-bounce ATR-FTIR employing a diamond crystal (Shimadzu IRTracer-100 with QATR accessory and DLATGS detector, the noise floor of the setup was estimated to 10^–4^ in blank measurements). ATR-FTIR spectra were collected under identical conditions with a spectral resolution of 4 cm^–1^ and 128–256 scans (128 scans for the isopropyl alcohol measurements, 256 scans for the LPS- and bacteria measurements; total measurement time was 150–300 s). Key regions, in particular 1250–1050 cm^–1^, were compared to evaluate sensitivity and consistency. For each bacterial sample, the measurement was repeated ten times and the ATR was cleaned between measurements.

The penetration depth with a diamond ATR crystal and a 45° angle of incidence is roughly a factor 4 greater than with the waveguide. At 1100 cm^–1^ it is 3.06 μm with the ATR and 0.72 with the waveguide (see the Supporting Information). The bacteria studied here are typically 0.5–2 μm in size, so there is some possibility that the difference in penetration depth will affect the spectra, e.g., by giving the cell membrane components somewhat greater weight in the waveguide spectra.

## Results and Discussion

The fabricated waveguide had good throughput down to ∼1050 cm^–1^ while maintaining high sensitivity. Below 1050 cm^–1^ the sputtered silicon film gave high losses. When evaluated with 10% isopropyl alcohol (Figure 4), the waveguide showed good consistency to ATR-FTIR and has approximately a factor of 20 higher sensitivity around 1100 cm^–1^. The difference in relative heights of the absorption peaks compared to ATR-FTIR is due to the sensitivity spectrum of the waveguide, which falls off quite quickly at higher wave numbers (Figure 2).

**Figure 4:**
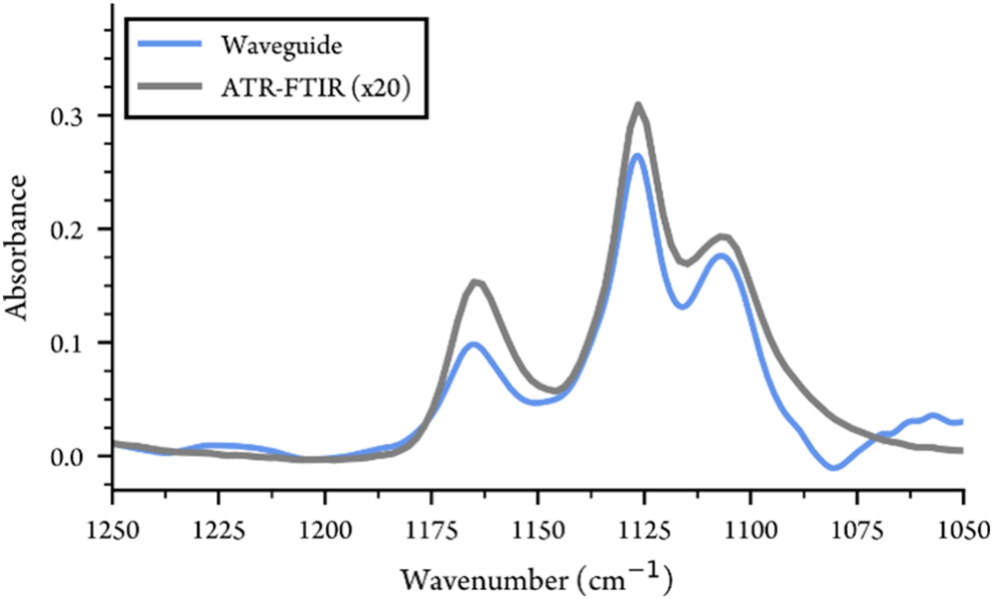
Waveguide spectrum of isopropyl alcohol (10%) with comparison to ATR-FTIR.

To demonstrate LPS measurement with this waveguide, we analyzed LPS solutions derived from two strains of *E. coli*. Measurements of the LPS solutions taken in regular time intervals during evaporation with the waveguide are shown in Figure 5A,B. The waveguide and the ATR-FTIR spectra were found to be consistent and the waveguide showed a significant absorption enhancement. As above, the relative peak heights are different between the ATR and waveguide due to the different sensitivity spectra. As the concentration increases during evaporation, the absorption increases. The main differences in the spectra of the two LPS molecules are observed at ∼1150 and 1060 cm^–1^. The region 1200–1050 cm^–1^ contains vibrational bands from phospholipids and carbohydrates, and may be difficult to accurately assign due to the complex and overlapping sugar modes.^48^ The band around 1225–1240 cm^–1^ is often assigned to the antisymmetric stretching of the phosphate double bond (P=O), while the symmetric stretching of the phosphate group is generally found around 1080–1100 cm^–1^.^49^ The high absorption observed at 1085 cm^–1^ for both LPS is however more likely due to sugar ring modes (involving C–O, C–O–C and C–OH vibrations) rather than phosphate vibrations.^50^ For LPS, the symmetric stretching of the phosphate group can instead be assigned to the high-wavenumber shoulder observed at ∼ 1110 cm^–1^.^50,51^ The band at 1150 cm^–1^ is assigned to complex sugar ring modes in the carbohydrate backbone and ester single bonds in the lipid A component of the LPS molecules.^22,48,50,51^ The higher absorption for LPS O111:B4 at 1150 cm^–1^, compared to LPS O26:B6, could reflect the presence of longer polysaccharide chains in LPS O111:B4, which is consistent with previous SDS-PAGE measurements.^52^ The band around 1060 cm^–1^ is attributed to complex sugar ring vibrations. The consistency between the waveguide and ATR-FTIR measurements is reduced toward 1050 cm^–1^, as the amount of light through the waveguide decreases significantly here, due to a combination of a fairly weak QCL source and losses in the sputtered Si film and undercladding.

**Figure 5:**
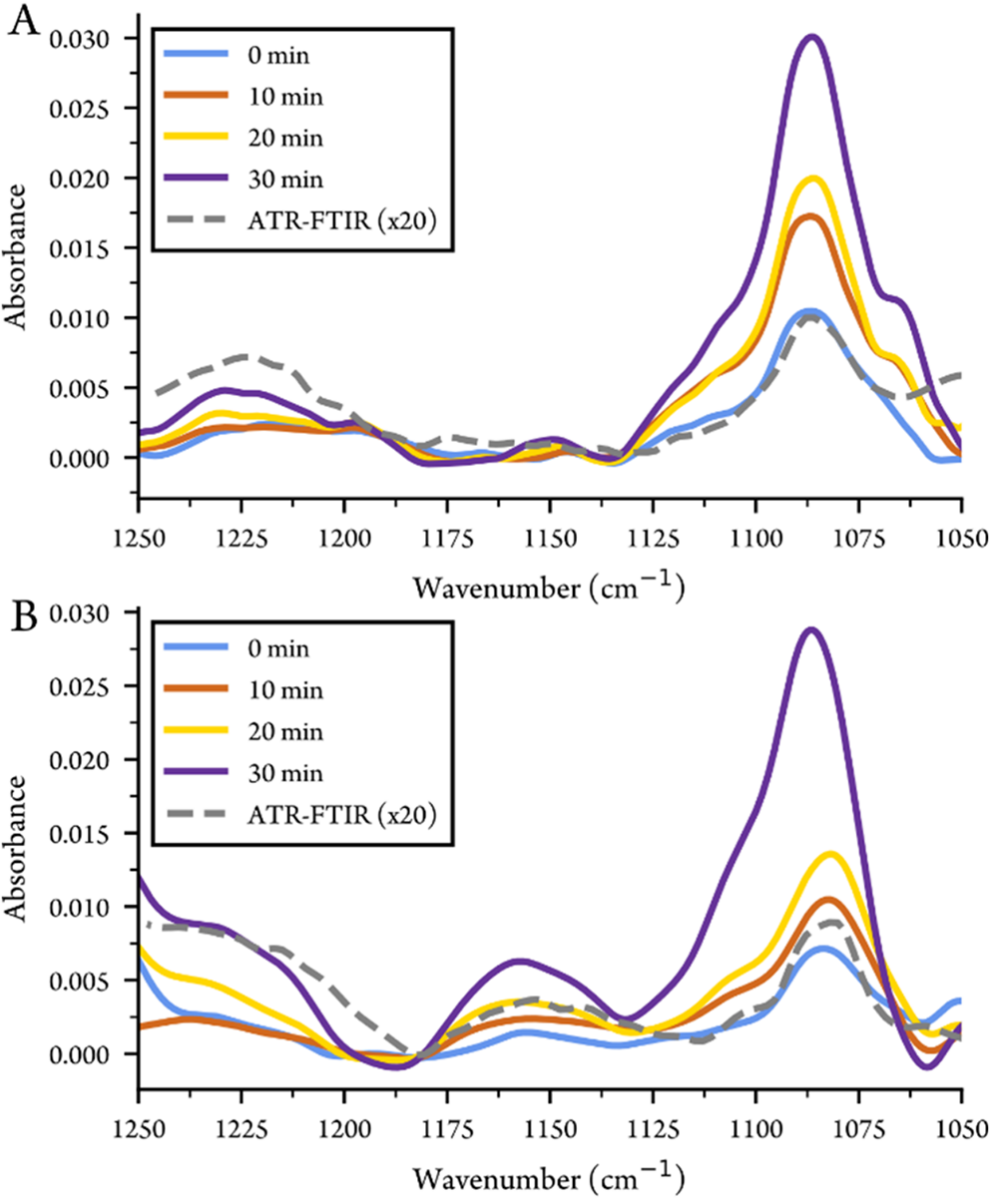
Waveguide spectra of (A) LPS O26:B6 and (B) LPS O111:B4 during evaporation with comparison to ATR-FTIR.

In order to get an estimate of the sensitivity and detection limit of the waveguide, each LPS was measured for concentrations between 1–5 mg/mL. The absorbance values at 1085 cm^–1^ for each LPS strain are shown in Figure 6 plotted against the concentration, together with linear fits to the data. We obtain a LOD of ∼0.15 mg/mL for LPS O26:B6 and ∼0.28 mg/mL for LPS O111:B4.

**Figure 6:**
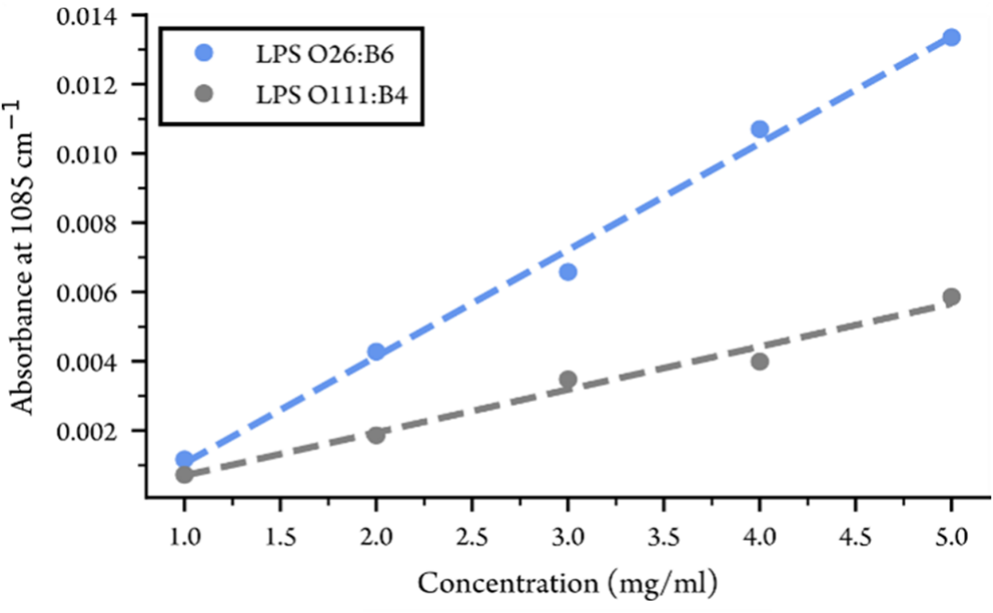
Absorbance values at 1085 cm^–1^ for LPS solutions with concentrations between 1–5 mg/mL.

Next, we evaluate the ability of the waveguide to characterize intact bacteria cells in aqueous solutions. In order to measure the full spectral range of the QCL system, i.e., between 1730–1050 cm^–1^, we let the concentration increase by evaporation for 40 min, as the waveguide exhibits lower sensitivity at higher wavenumbers. After 40 min most liquid is dried out, but the bacteria are still in a hydrated state and water is used as reference measurement. By optimizing the waveguide for the 1200–1050 cm^–1^ range, we achieve high sensitivity in the region of highest interest while maintaining enough signal at the strong water absorption around 1640 cm^–1^ to measure the amide I peak. With a waveguide that has high sensitivity at all wavelengths, water would completely absorb the signal in a wide band around 1640 cm^–1^.^40^

Figure 7 shows a representative waveguide spectrum of *E. coli* between 1730 and 1050 cm^–1^. The three characteristic amide peaks at 1650 cm^–1^, 1540 cm^–1^ and 1240 cm^–1^ are visible and the peak due to phospholipids, nucleic acids and polysaccharides at 1085 cm^–1^ dominates the spectrum. We also observe absorptions around 1450 and 1400 cm^–1^, due to CH_2_ and CH_3_ bending in proteins and lipids, and C=O stretching in the carboxylate groups of amino acids.^48,49^ The discontinuities in the spectrum are due to the shifts between the QCL ranges. The green curve in Figure 7 shows the expected relative sensitivity between the waveguide and ATR measurements (see the Supporting Information). The difference in sensitivity is clear from how the relative strengths of the absorptions differ between the two measurements. For example, the peak at 1086 cm^–1^ is smaller than the amide I and II peaks in ATR measurements, while the peak in waveguide sensitivity makes it the dominant feature in the waveguide spectrum. A summary of the absorption bands reported in this work is presented in Table 1.

**Table 1:** Assignments of IR Absorption Bands Reported in This Work^22,48–50,53^

Wavenumber	Assignment
1650 cm^–1^	Amide I band (protein structure)
1540 cm^–1^	Amide II band (protein structure)
1450 cm^–1^	CH_2_ and CH_3_ bending of proteins and lipids
1400 cm^–1^	C=O stretching of carboxylate groups in nucleic acids
1250 cm^–1^	Amide III (protein structure)
1220–1250 cm^–1^	P=O antisymmetric stretching of > PO
∼1150 cm^–1^	C–O stretching in esters and glycosidic linkages
∼1120 cm^–1^	C–O, C–C and C–O–C stretching in carbohydrates
∼1110 cm^–1^ and ∼1080 cm^–1^	P=O>PO_2_^–^ symmetric stretching of
∼1080 cm^–1^ and ∼1060 cm^–1^	Complex sugar ring modes

**Figure 7:**
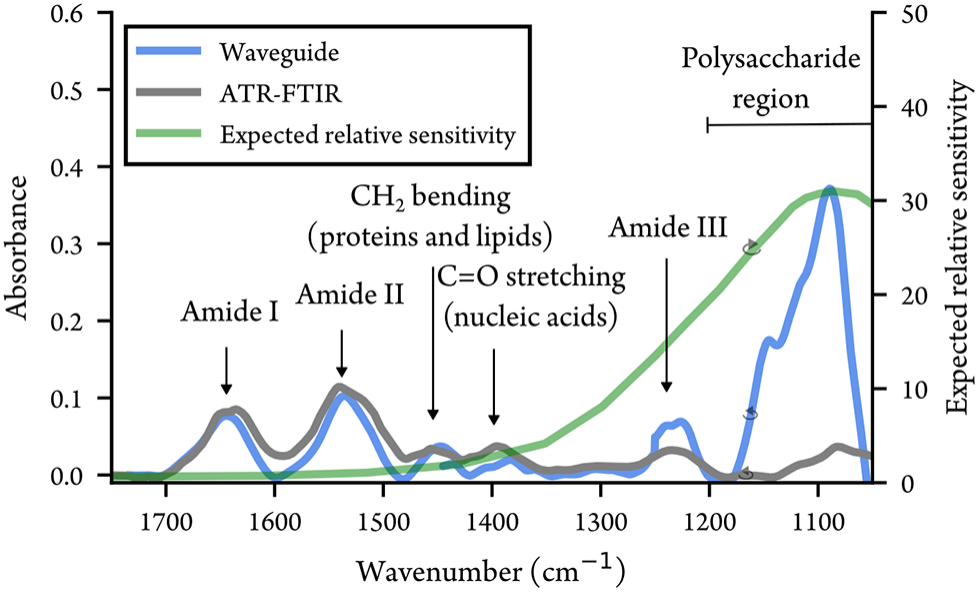
Waveguide and ATR spectra in the full spectral range of our source taken of *E. coli* cells in water after drying. The main absorption features are highlighted. The green line shows the expected sensitivity curve of the waveguide compared to the ATR.

For the range 1250–1050 cm^–1^, the measurements were performed without evaporation and the measurement was repeated several times over 2 days for each bacterium (12 times for *S. aureus* and 8 for *E. coli*) to evaluate the repeatability. Figure 8A,B show the mean and standard deviations of the measurements taken with the waveguide and ATR-FTIR. When measuring whole bacteria cells, many different cell components (including for example the cell wall, cytoplasmic membrane and nucleic acids) contribute to the spectrum, but since the region 1200–1050 cm^–1^ is dominated by carbohydrate and phosphate vibrations, we expect large spectral contributions from the bacterial cell wall. The major absorptions observed in the spectra occur around 1230, 1160, 1120, and ∼1080 cm^–1^. The band at 1225–1250 cm^–1^, is attributed to antisymmetric stretching of the PO_2_^–^ group in phosphate and phosphodiesters, as well as the amide III band, while the bands around 1160 and 1120 cm^–1^ are attributed to C–O stretching of glycosidic linkages and C–O–C stretching of carbohydrates, originating from the bacterial cell wall.^51,53,54^ The band around 1080 cm^–1^ dominating the spectrum for both bacteria, is assigned to the symmetric stretching vibrations of the PO_2_^–^ group present in nucleic acids, LPS and teichoic acids, as well as complex sugar ring modes of cell wall components.^53^ The standard deviations shown in Figure 8 show that while the absolute variations between spectra are higher for the waveguide, the high absorbance still gives a better ratio of absorbance to deviation at the enhanced absorption peaks. For the lowest wavenumbers, close to 1050 cm^–1^ the noise level in the waveguide measurements increases dramatically due to the weak signal through the waveguide.

**Figure 8:**
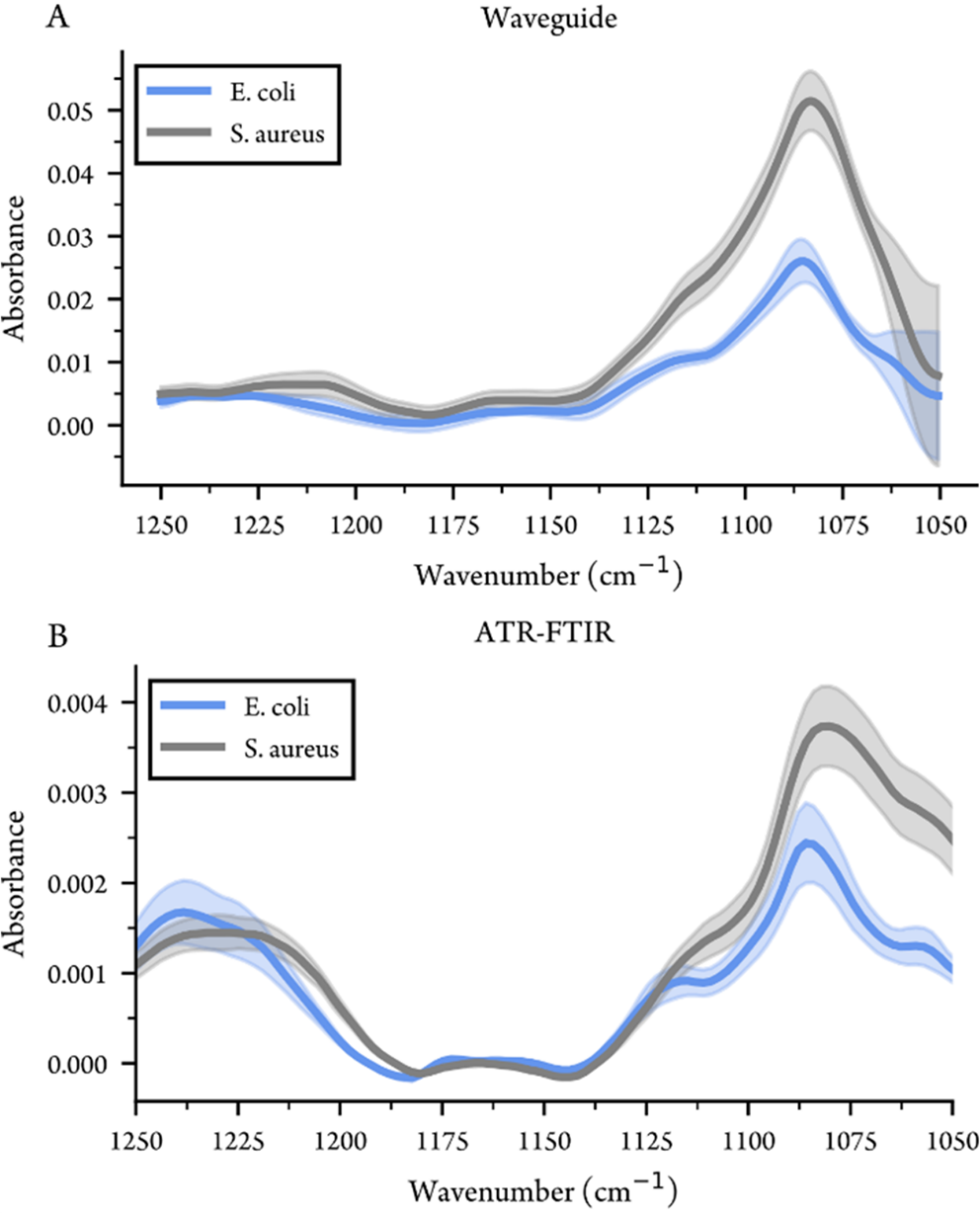
*S. aureus* and *E. coli* measured in water with (A) waveguide and (B) ATR-FTIR in the region 1250–1050 cm^–1^. Solid lines show the mean value of the measurements, while the shadowed area is the standard deviation.

PCA analysis was performed in the range 1250–1070 cm^–1^ for 20 spectra collected with the waveguide (12 for *S. aureus* and 8 for *E. coli*). The result of the PCA analysis is presented in Figure 9. Figure 9A shows the distribution of the measurements when projected to the first two principal components. The measurements are separated into two clusters corresponding to the different bacteria by the first principal component, while the second principal component does not seem to capture much of the variability between the two classes of bacteria and mainly represents measurement noise. By inspection of the loading vector of the first principal component, shown in Figure 9B, the most important wavenumbers can be seen to be are located at 1239, 1151, 1126.5, 1091 and 1077 cm^–1^. The peak at 1239 cm^–1^ corresponds to the amide III band and antisymmetric > P=O stretching vibration, and the relatively increased absorption for *E. coli* could be explained by the thinner cell wall, leading to increased amide contributions from nucleic acids. The bands around 1151 cm^–1^ and 1126.5 cm^–1^ are attributed to glycosidic linkages and carbohydrate vibrations in the cell wall’s sugar components,^53^ while the peaks at 1190 and 1176 cm^–1^ are related to the difference in peak position and shape of the main absorption around 1080 cm^–1^.

**Figure 9:**
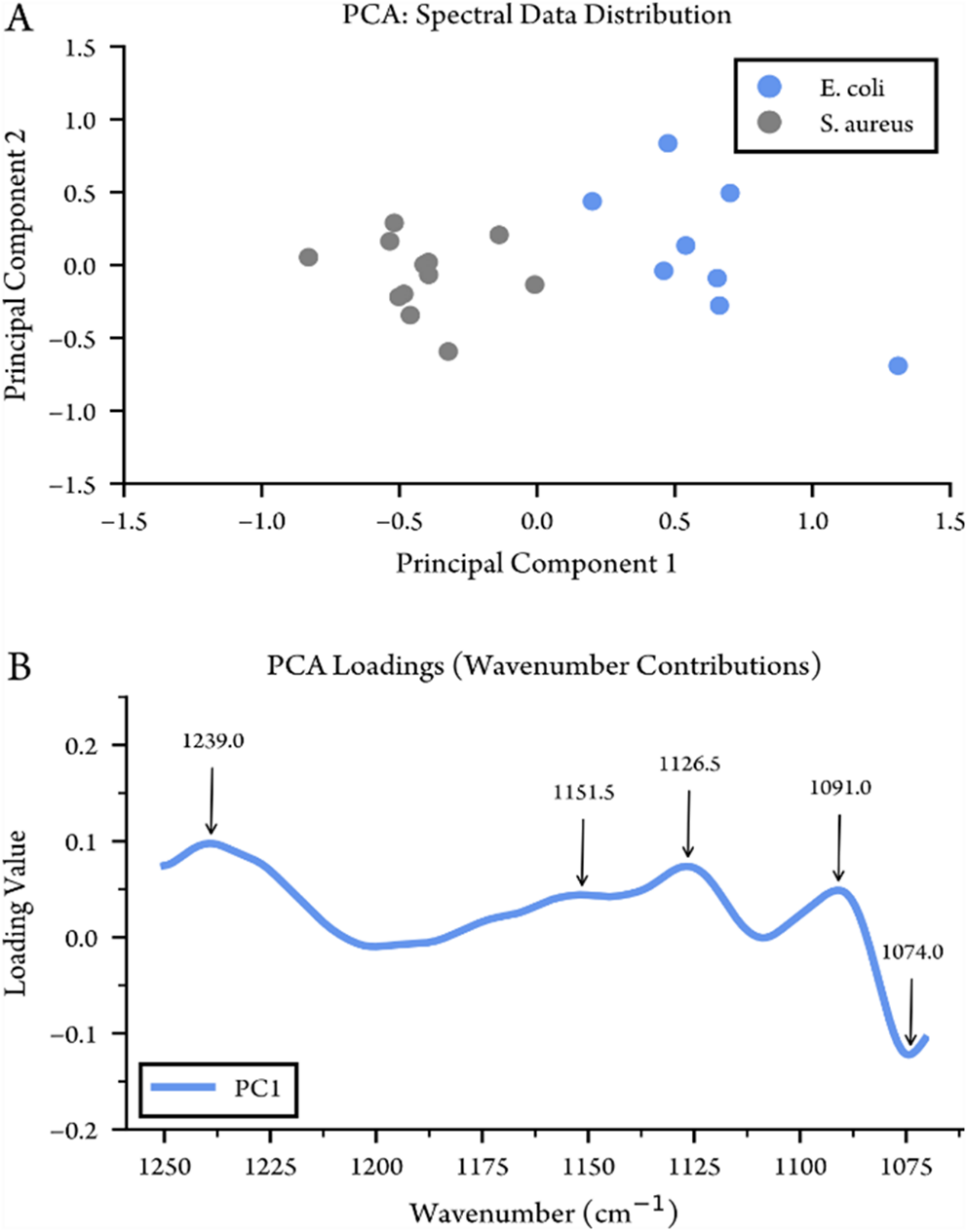
Principal component analysis of the waveguide spectra of intact bacteria cells in water. (A) 2D scores plot shows the distribution of the individual sample spectra for *S. aureus* (blue) and *E. coli* (gray). (B) The loading vectors of the first principal component highlights the variation in the spectra over the wavenumbers.

The main difference among the bacteria studied here lie in the composition of the bacterial cell wall. The observed spectral differences can therefore be linked to the presence of LPS as well as the absence of teichoic acids in *E. coli* with respect to *S. aureus*, and the much thicker peptidoglycan layer of *S. aureus*.^53^ In particular, the positive absorption contributions around 1150 and 1126 cm^–1^ in the spectrum of *E. coli* could be linked to the presence of LPS in the cell wall. The spectral position of the phosphate and carbohydrate absorption around 1080–85 cm^–1^, and the ratio of the magnitude of the absorption bands of e.g., amide III and the band around 1080 cm^–1^, are also distinct, making it straightforward to distinguish between the two bacteria. The ATR-FTIR spectra are consistent with those obtained with the waveguide (the waveguide spectra are skewed toward lower wavenumbers due to the local sensitivity enhancement). We do not see any absorptions that are unique to the waveguide spectra and not seen in the ATR measurements, as previously reported.^37,38^ While higher spectral resolution may sometimes allow better separation of overlapping absorptions, the underlying absorption mechanism, the interaction between the electromagnetic field and molecular vibrations, is the same with a waveguide as with an ATR element and the same absorptions should be accessible to both. We do see an order of magnitude increase in absorbance in the optimized range with this 5 mm long waveguide, compared to single reflection ATR-FTIR measurements. With improved QCL stability^55^ and measurement setup, it should be able to collect equivalent spectra with the same order lower concentration. This would make it similar in sensitivity to a 10–20 reflection ATR accessory,^56^ but smaller and requiring much lower sample volume.

## Conclusion

Differentiation between bacterial LPS and intact bacteria cells by MIR waveguide spectroscopy was demonstrated in aqueous solution. The differences in the spectra of *E. coli* and *S. aureus* were mainly attributed to the large differences in cell envelope between Gram-positive and Gram-negative bacteria. Further studies on a wide range of both Gram-positive and Gram-negative bacteria will be needed to make a general prediction, however. By specifically optimizing the waveguide sensitivity in the region 1200–1050 cm^–1^, we showed more than an order of magnitude enhancement of the MIR absorptions compared to ATR-FTIR. This waveguide combines high sensitivity in the polysaccharide region with low sensitivity at the amide I band, where the signal can otherwise be lost in aqueous solution due to the strong water absorption at 1640 cm^–1^. We will further optimize this sensitivity spectrum in the future, to get as much data as possible out of a single measurement. We are also working on further improving the waveguide geometry and measurement setup stability to improve repeatability and reduce noise and stray light on the detector, which will improve the SNR. Improving the quality of the silicon film should reduce losses at lower wavenumbers, extending the useful range of the waveguide further. Diamond waveguides can further be surface modified to specifically enrich the target species,^57^ which should make it possible to drastically increase the sensitivity of the measurement and allow high-quality studies of more complex biofluids such as e.g., blood or saliva. Compared to standard ATR-FTIR instruments, waveguide platforms are more sensitive to instability and noise, but also offer the advantage of higher sensitivity per unit length, as well as the potential for miniaturization, integration with microfluidics, and lower cost.

## Supplementary Material



## Notes

In depth exploration of the waveguides modes when applying a high index film on a multimode waveguide (PDF)
