# Supplementary material for: Waveguide Spectroscopy for Differentiation of Bacteria
Source: Anal Chem. 2025 Sep 26;97(39):21314–23. doi: 10.1021/acs.analchem.5c02661 (PMC12509186; doi:10.1021/acs.analchem.5c02661)
Supplement: Supplementary file 1 [file ac5c02661_si_001.pdf]

Supporting Information

Waveguide Spectroscopy for Differentiation of Bacteria

Arvid Angelsten, Pontus Forsberg, Håkan Engqvist, Wei Xia, and Mikael Karlsson*

Affiliation:
Department of Materials Science and Engineering, Uppsala University, 751 03 Uppsala, Sweden

*Corresponding author; Email: mikael.karlsson@angstrom.uu.se

Abstract

This appendix will explore in more depth what happens with the modes and sensitivity of a diamond film waveguide when a silicon film is applied on top.

Table of content

Figure S1. The mode profiles of all 8 TE modes at 9 µm wavelength.
Figure S2. Penetration depth of the first 8 TE modes in the diamond waveguide and comparison to a diamond ATR at 45° incidence angle.
Figure S3. Proportion of power above the waveguide for 8 TE modes.
Figure S4. Profiles at several wavelengths of the first four TE modes with a silicon film on top of the waveguide.
Figure S5. Mode profiles for all 8 TE modes at 9 µm wavelength with a 410 nm thick silicon film on the waveguide.
Figure S6. Penetration depth with a silicon film on the waveguide for 8 TE modes.
Figure S7. Proportion of power above the waveguide for the 8 TE modes.
Figure S8. Simulated geometry and snapshot of the propagated field with 9 µm wavelength.
Figure S9. Averaged squared E-field along the waveguide with silicon film with 9 µm wavelength.
Figure S10. Fitting mode profiles to the propagated profile.
Figure S11. Proportion of modes fitted to the propagated field.
Figure S12. Expected sensitivity curve for the waveguide.
Figure S13. Effective thickness comparison for the waveguide and FTIR-ATR experiments.

The total waveguide thickness here is fixed at 19.41 µm. Where a silicon film is included, the thickness of this layer is 410 nm, matching the measured thickness of the film on the waveguide used in the paper. The wavelength range under consideration is between 5.5 and 11.5 µm (1818 cm^{-1} to 870 cm^{-1}). For a similar study, but with a varying film thickness at a fixed wavelength, see [1]. We focus on the TE modes here as they are more strongly affected by a thin film. The calculations here do not take absorption into account and are only really accurate for weakly absorbing media.

The mode profile calculations here are made with a simple finite difference mode solver implemented in MATLAB. The refractive indices used were fixed for diamond (2.38) [2] and the sputtered silicon (3.53) [3], while those for water (as the analyte) and AlN (the undercladding) were taken from [4] and [5] respectively. In our previous work we found that the sensitivity peaked for a thinner film than we expected [1]. This was most likely due to the sputtered amorphous Si film having a higher refractive index than the value for crystalline Si that was used for those simulations. Here we therefore use the refractive index of hydrogenated amorphous Si (~3.53 in our wavelength range) measured by Franta et al [3]. Our Si film is not hydrogenated, but since we get good agreement with our previous experimental results [1] by using this value, it is likely close enough for our purposes.

Without the silicon film, the diamond is a simple slab waveguide. There are 8 TE modes that can be supported in the whole wavelength range (10 for the shortest wavelengths). As the refractive indices above and below the waveguide are both well below that of diamond, the waveguide is almost symmetric and so the modes are nearly symmetric or antisymmetric. Their profiles at 9 µm are plotted in figure S1.

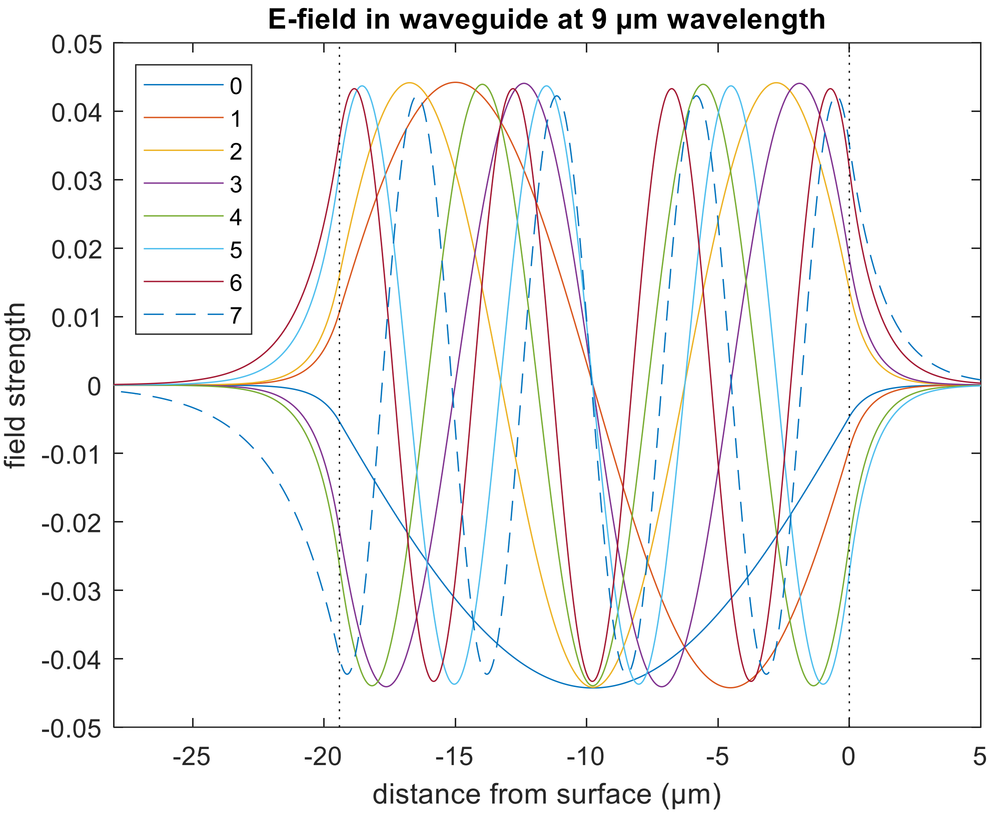

Figure S1. The mode profiles of all 8 TE modes at 9 µm wavelength.

The x axis here shows the distance from the upper surface of the waveguide and the dotted lines show the interfaces. The thickness of the diamond film is 19.41 µm. There are four symmetric and four antisymmetric modes. Outside the waveguide the field amplitudes fall off exponentially with distance (z) as $E = E_0 \, exp(-z/d_p)$ where E_0 is the field strength at the surface and d_p is the penetration depth. The penetration depth is greater in the undercladding than in the analyte due to the higher refractive index on this side. The penetration depth in the analyte is roughly linear with wavelength for the lower modes (figure S2).

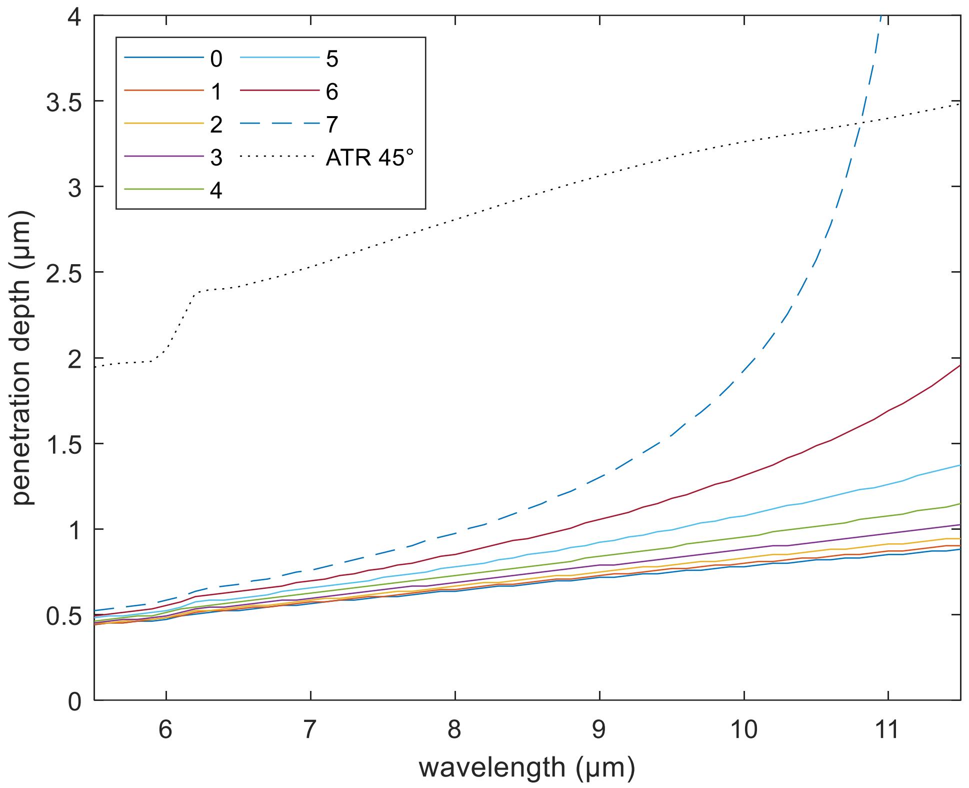

Figure S2. Penetration depth of the first 8 TE modes in the diamond waveguide and comparison to a diamond ATR at 45° incidence angle.

For comparison, the penetration depth of a diamond ATR element with a 45° incidence angle is included in figure S2, given by $d_p = \lambda / (2\pi\ (sin^2\theta - n_{21})^{1/2})$, where λ is the wavelength, θ the incidence angle and n_{21} the refractive index ratio between water and diamond. The bend close to 6 µm is due to the refractive index of water changing quite rapidly around the absorption peak there. Note that the penetration depth alone does not determine the interaction strength, merely how quickly the field decays with distance from the surface. It is however a good measure for how surface sensitive the measurement is.

To get a measure for how effectively a mode can interact with the analyte, we integrate the squared field above the waveguide and divide it with the total squared field. This gives us the proportion of power flux in the analyte. Since this is the proportion of the field that can interact and be absorbed by the analyte, it is proportional to the sensitivity per unit length of the waveguide. Plotting it against wavelength for the eight modes gives us figure S3.

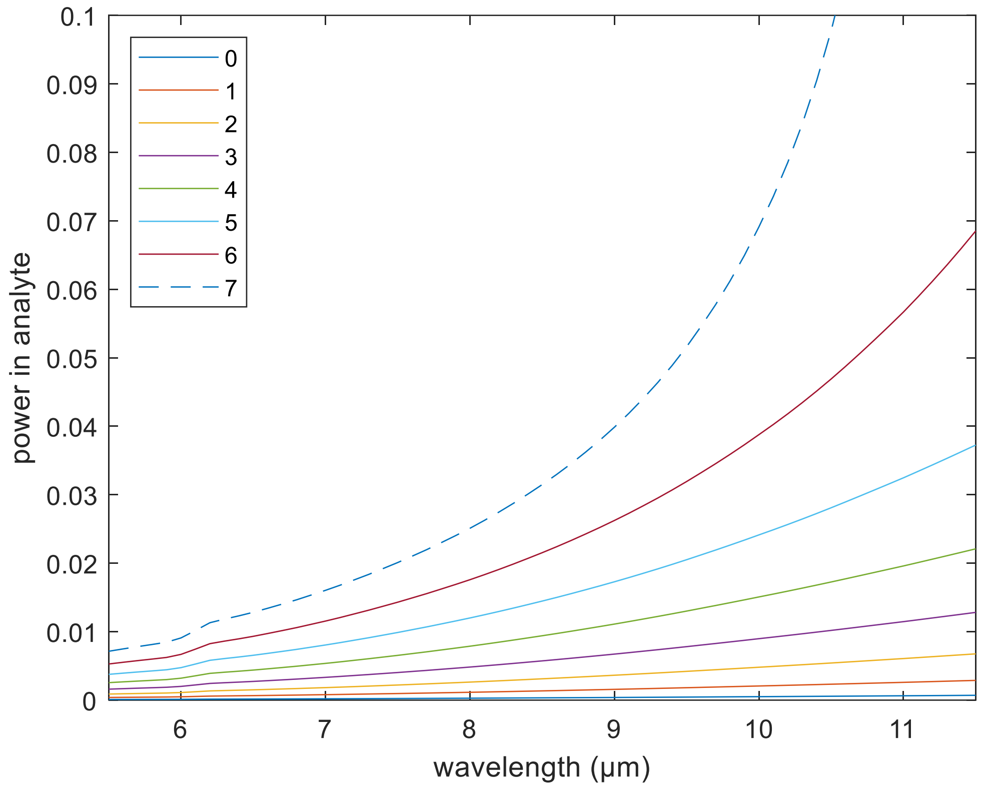

Figure S3. Proportion of power above the waveguide for 8 TE modes.

The higher modes have a large proportion of the power flux in the analyte and can interact strongly. Unfortunately, the proportion in the undercladding is even greater (as we saw in figure S1) and since the undercladding is somewhat absorbing this reduces the amount of light through the waveguide. In preliminary experiments we did not get any light through a waveguide at longer wavelengths when we attempted to couple into the higher modes.

In the experiment described in the paper, the waveguide has end faces that are perpendicular to the propagation direction and light is coupled straight into the waveguide by focusing with a cone angle of a few degrees. In this configuration we are most likely to couple light into low symmetric modes, since the input is symmetric and the higher modes have highly tilted phase fronts that do not match the input.

When a silicon film is applied on the surface of the waveguide the behaviour of the modes depends strongly on the thickness of the film in relation to the wavelength. Here we show results with a 410 nm thick film, which is the measured thickness on the waveguide used in the paper. To reduce clutter figure S4 shows the mode profiles for only the lowest four modes at six wavelengths.

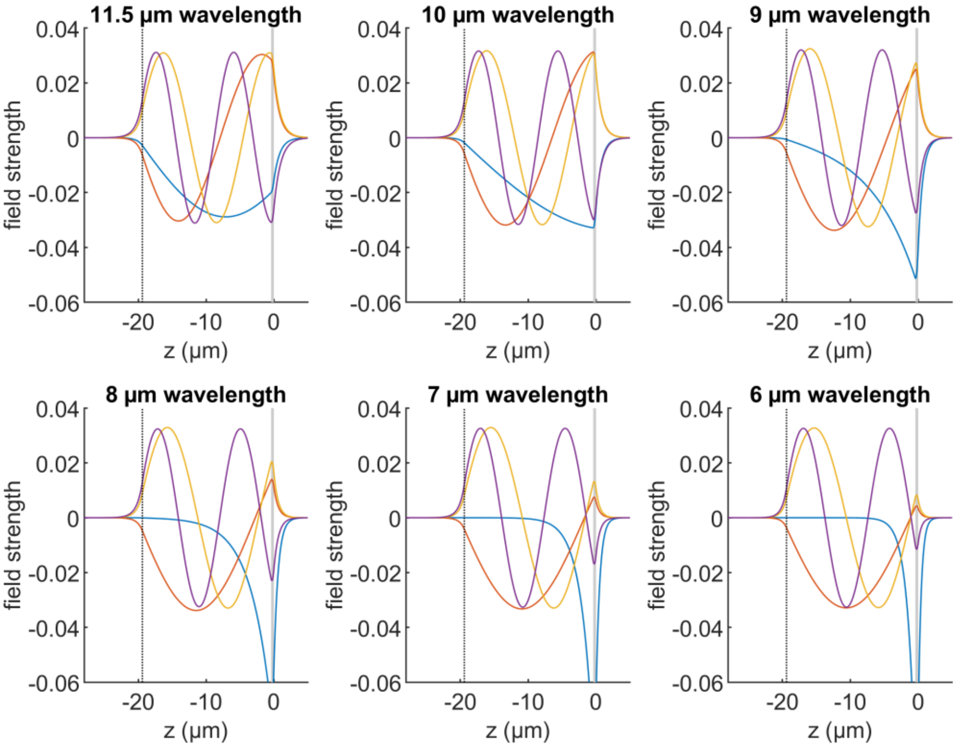

Figure S4. Profiles at several wavelengths of the first four TE modes with a silicon film on top of the waveguide.

At long wavelengths the film affects the profiles by skewing them towards the surface, but their shape remains largely the same. As the wavelength decreases, the lobe closest to the surface for each mode becomes more and more confined to the Si film. This is most dramatic for the lowest mode since it has only one lobe. At some wavelength the lowest mode is confined to the film as a mode in a silicon on diamond waveguide. The remaining modes “lose” one lobe to the silicon film, which shifts their symmetry in the thicker diamond layer, the antisymmetric modes become nearly symmetric and vice versa.

Figure S5 shows all eight mode profiles at 9 µm wavelength, for comparison with figure S1.

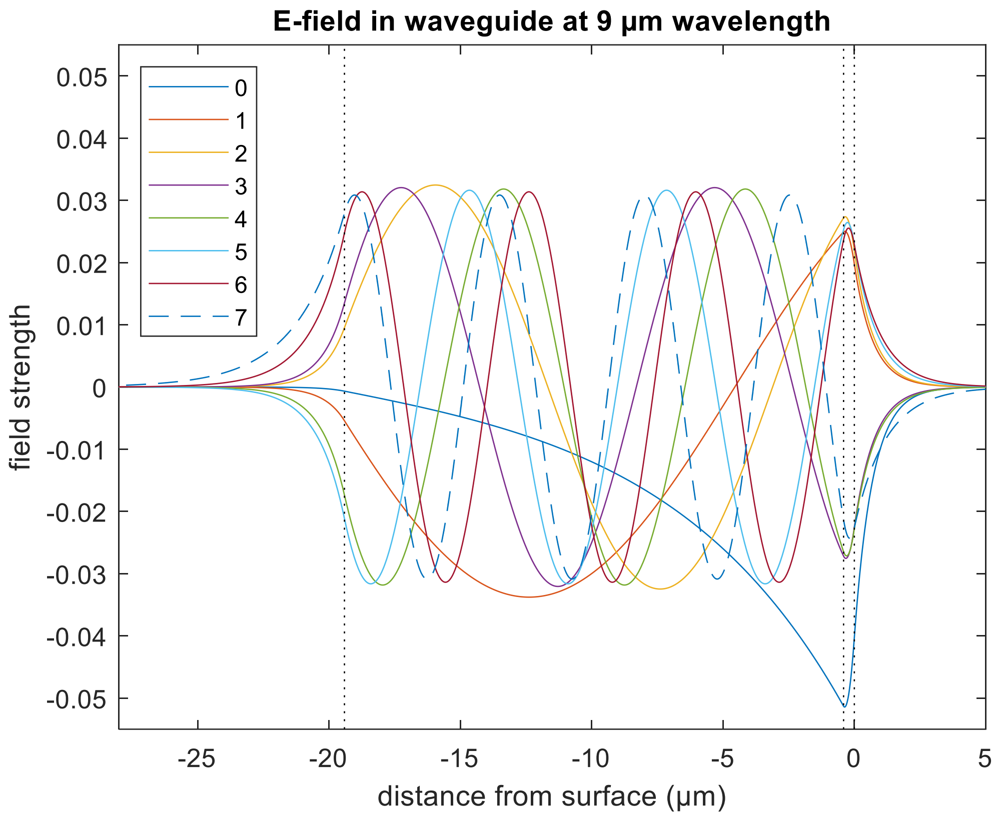

Figure S5. Mode profiles for all 8 TE modes at 9 μm wavelength with a 410 nm thick silicon film on the waveguide.

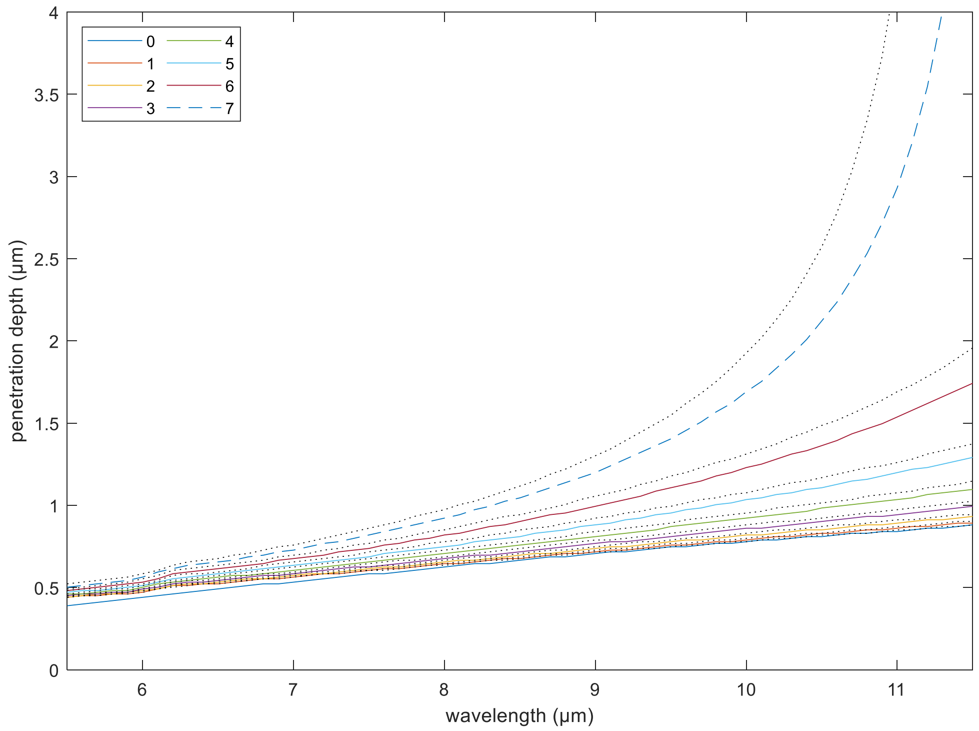

Figure S6. Penetration depth with a silicon film on the waveguide for 8 TE modes. The dotted lines show the penetration depth curves without a silicon film, as shown in figure S2.

Despite this dramatic change in the mode profiles, the penetration depth is nearly unchanged from the case with no film on the waveguide (figure S6). It is a little bit lower, but mainly for the higher modes.

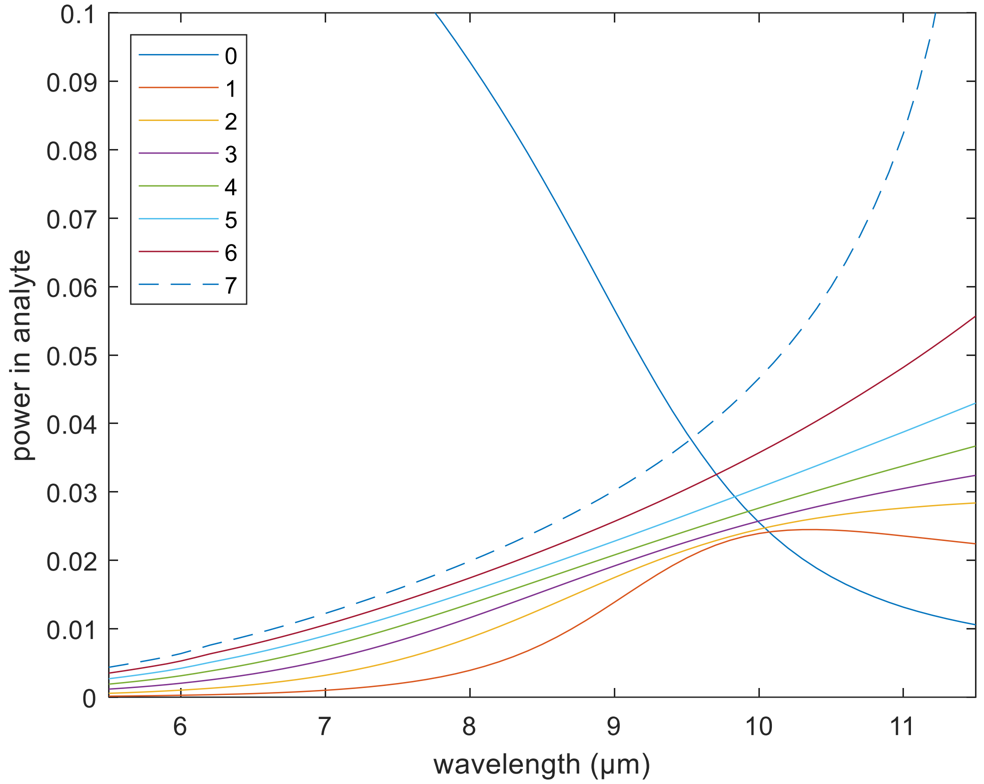

Figure S7. Proportion of power above the waveguide for the 8 TE modes.

Figure S7 shows the proportion of power flux above the waveguide with a silicon film (to be compared with figure S3). Here we see a dramatic change due to the Si film. The evanescent field in the analyte is enhanced for all modes, and the curves for lowest modes change shape. The TE0 mode changes behaviour most dramatically, becoming high at low wavelengths, which is not surprising considering the shift in the mode profile we saw in figure S4. The TE1 mode has a peak close to 10 μm that coincides with the point where it intersects the TE0 mode. This occurs at the wavelength which can just barely be confined as a mode in the silicon film. From our previous work [1] we expect the peak in sensitivity for the waveguide to be close to this wavelength. Since mode calculations are quick and easy, finding the film thickness that can barely support a mode with the wavelength where one wants peak sensitivity is a good starting point for design. As we shall see below, peak sensitivity will fall a little below this wavelength.

As mentioned above, the geometry of our experiment favours coupling into low symmetric modes. From this we would expect the dominant mode in the waveguide with a silicon film to be the TE0 mode at long wavelengths (similar to the waveguide with no film). At short wavelengths, where the TE0 mode is strongly confined to the surface and the TE1 mode is nearly symmetric, the TE1 mode should be the dominant one. In order to explore the transition between these extremes, we turn to Finite Difference Time Domain (FDTD) simulations to propagate the field. For this we used the simulation package MEEP (MIT Electromagnetic Equation Propagation) in 2 dimensions. The simulated geometry is shown in figure S8. The first 700 μm of the waveguide were simulated, with the first 150 μm without a silicon film (this to match the experiment, where the edges of the

waveguide were masked during silicon sputtering). A Gaussian beam with a 5 µm beam radius was used as the input. The spatial resolution of the simulation was 40 nm, which is much smaller than the thickness of the Si film. Variations in film thickness smaller than the resolution were taken into account by subpixel smoothing in MEEP.

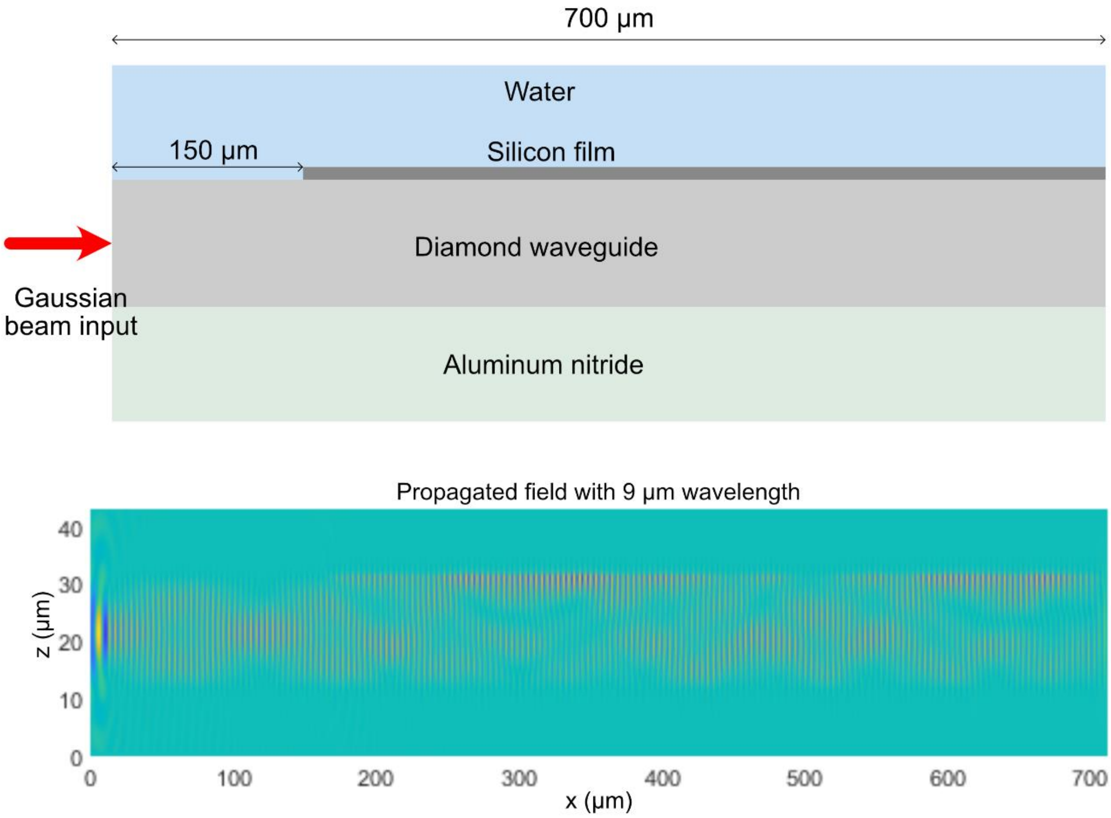

Figure S8. Simulated geometry and snapshot of the propagated field with 9 µm wavelength.

In the lower part of figure S8 a snapshot of the propagated Ey field is shown for 9 µm wavelength. We note that in the first part of the waveguide, without a silicon film, the field in the waveguide looks like the TE0 mode. In the latter part of the waveguide, the mode picture is more complex, but it is clear that the field is stronger close to the surface with the silicon film.

To compare the profile of the propagated field with the modes, we average the squared field in figure S8 along the propagation direction (x in the figure) for a full beat length between the TE0 and TE1 modes (figure S9).

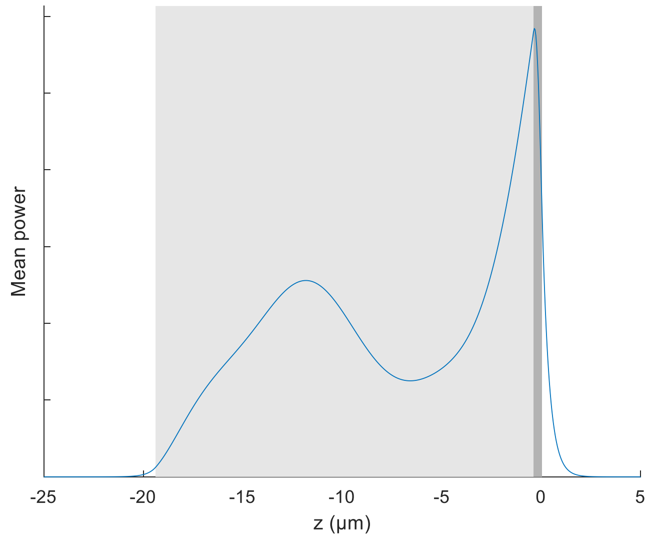

Figure S9. Averaged squared E-field along the waveguide with silicon film with 9 µm wavelength.

Comparing this with the mode profiles in figure S5, it looks like the main components are the TE0 and TE1 modes as expected. To get a better idea of the proportions of the different modes, we fit a combination of the squared profiles of the first four TE modes to the curve in figure S9. Figure S10 shows the result with the propagated profile as a solid black and the fitted profile dashed green. The four modes used for the fit are plotted in the proportions they are included in the fit. At 9 µm wavelength this was 42.4% TE0, 49.7% TE1, 1.4% TE2 and 6.5% TE3.

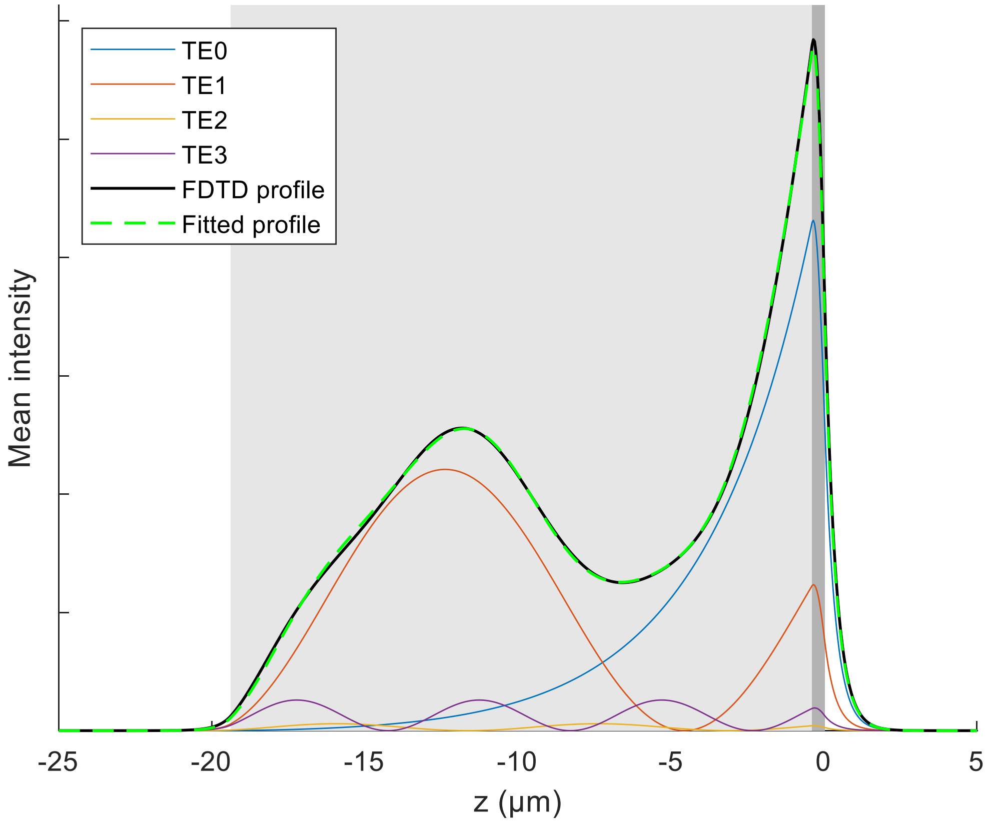

Figure S10. Fitting mode profiles to the propagated profile.

Repeating this for several wavelengths, we can plot the proportions of modes against wavelength in figure S11. To verify that the subpixel smoothing in MEEP gives the expected effective thickness of the film, mode profiles with small variations in film thickness were fitted to the result. The overall best fit was for 409 nm, while the nominal simulated film thickness was 410 nm, so the averaging worked very well.

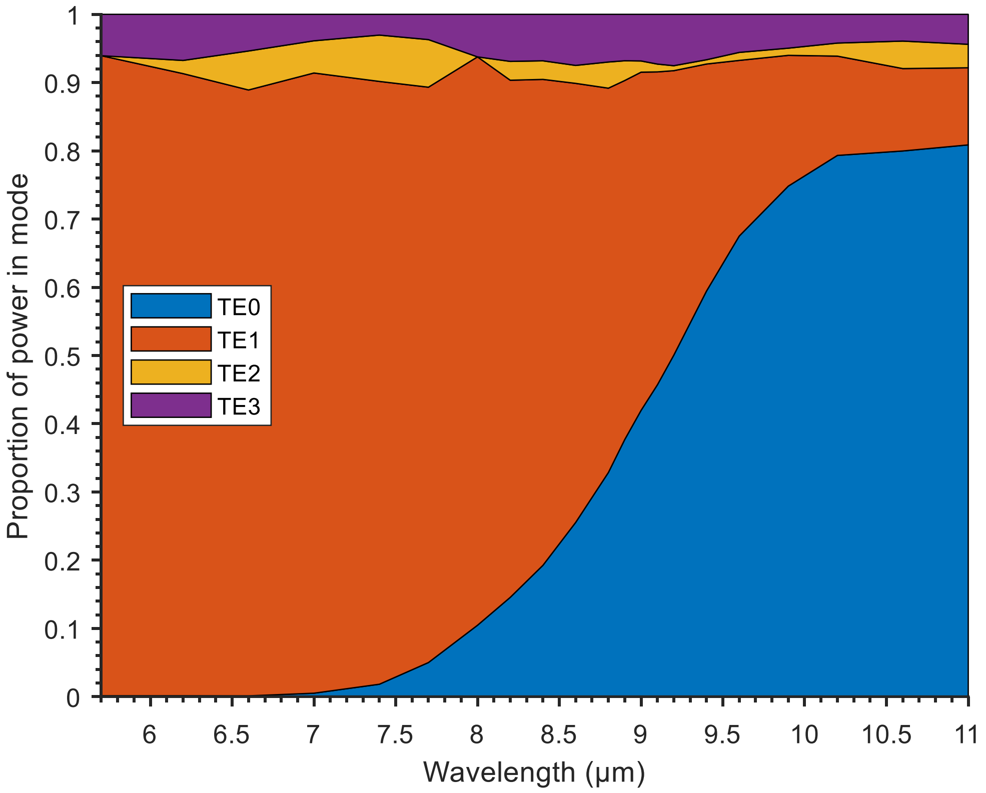

Figure S11. Proportion of modes fitted to the propagated field.

Finally, we can sum up the proportion of power flux above the waveguide (figure S7) according to these proportions to estimate how strongly the field in the waveguide can interact with the analyte across the wavelength range (figure S12) (this gives a more stable result than extracting this power directly from the FDTD simulation result, due to the limited resolution leaving the positioning of the surface somewhat uncertain).

The simulations do not exactly match the experimental conditions, in particular the shape of the beam from our QCL source is not perfectly Gaussian and changes somewhat with wavelength. The positioning of the sensitivity peak in figure S12 is also sensitive to the thickness and refractive index of the silicon film, both of which are associated with some uncertainty. This means that while we expect the experiment to qualitatively follow the curve in figure S12, some deviation of the peak position is likely.

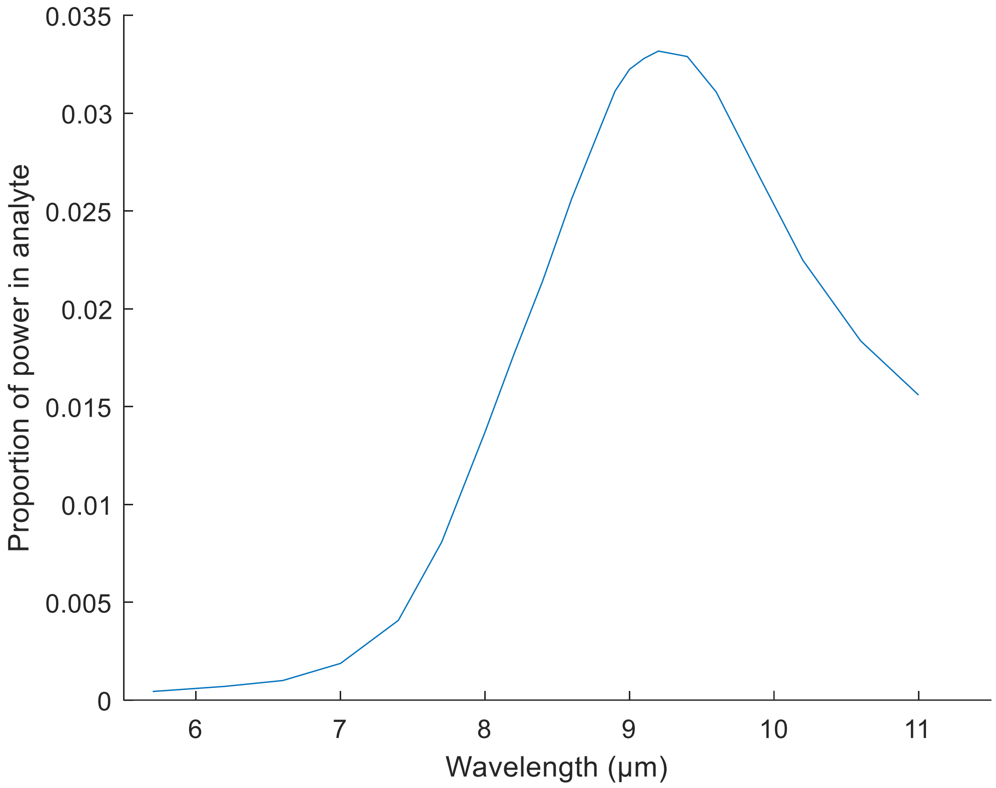

Figure S12. Expected sensitivity curve for the waveguide.

Multiplying with the length of the waveguide we get the "effective thickness", the thickness that should give the same absorption in a transmission experiment following the Beer-Lambert law. Comparing this to the effective thickness of a diamond ATR with 45° incidence (using the formulas in [6]) we can estimate the expected absorbance in the waveguide experiment compared with ATR.

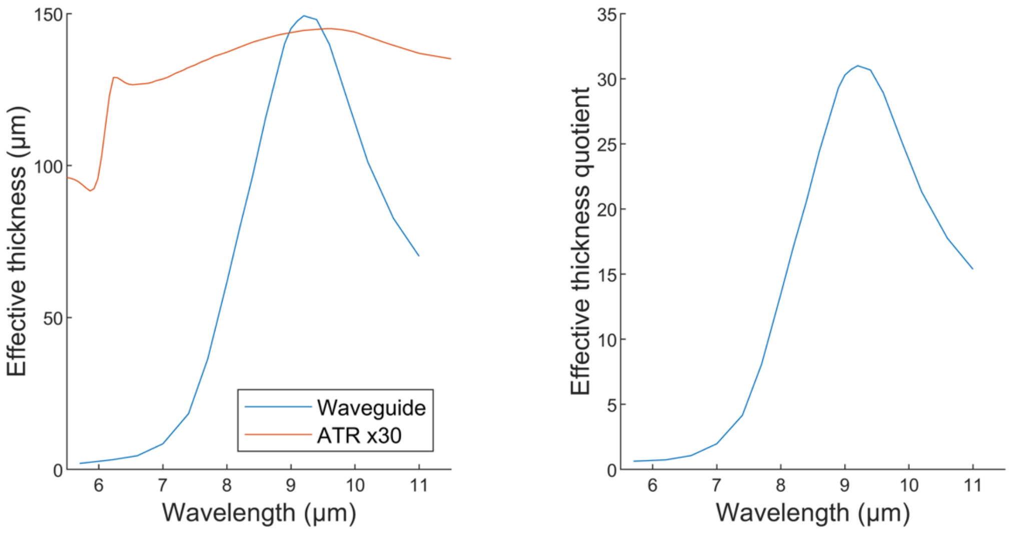

Figure S13. Effective thickness comparison for the waveguide and FTIR-ATR experiments. On the left the calculated effective thicknesses (the ATR curve has been multiplied by 30 for visibility) and on the right the Waveguide effective thickness divided by that of the ATR.

In figure S13 we see that we expect the waveguide sensitivity to vary from ~0.7 times the ATR at the short wavelength end to a peak of 31 times as sensitive.

The experimental results in the article roughly follow this curve, but the waveguide was actually closer to 20 times as sensitive at the peak wavelength calculated here. The discrepancy may be ascribed to both limitations of these simplified calculations and errors in the input, in particular the refractive index values of the sputtered films are uncertain.

References

[1] Forsberg, P.; Perez, L.; Karlsson, M. Enhancing sensitivity of mid-infrared waveguide spectroscopy with a high-index thin film. *ACS Applied Optical Materials* **2023**, *1* (2), 536–543. https://doi.org/10.1021/acsaom.2c00108.

[2] Zaitsev, A. M. *Optical Properties of Diamond*; Springer Berlin Heidelberg: Berlin, Heidelberg, 2001.

[3] Franta, D.; Nečas, D.; Zajíčková, L.; Ohlídal, I.; Stuchlík, J. Advanced modeling for optical characterization of amorphous hydrogenated silicon films. *Thin Solid Films* **2013**, *541*, 12–16. https://doi.org/10.1016/j.tsf.2013.04.129.*

[4] Hale, G. M.; Querry, M. R. Optical constants of water in the 200-nm to 200-µm wavelength region. *Applied Optics* **1973**, *12* (3), 555. https://doi.org/10.1364/ao.12.000555.*

[5] Kischkat, J.; Peters, S.; Gruska, B.; Semtsiv, M.; Chashnikova, M.; Klinkmüller, M.; Fedosenko, O.; Machulik, S.; Aleksandrova, A.; Monastyrskyi, G.; Flores, Y.; Ted Masselink, W. Mid-infrared optical properties of thin films of aluminum oxide, titanium dioxide, silicon dioxide, aluminum nitride, and silicon nitride. *Applied Optics* **2012**, *51* (28), 6789. https://doi.org/10.1364/ao.51.006789.*

[6] Harrick, N. J and du Pré, F. K. Effective thickness of bulk materials and of thin films for internal reflection spectroscopy. *Applied Optics* **1966**, *5* (11), 1739.

*Numerical values for the refractive indices can be found at:
M. N. Polyanskiy, "Refractive index database," https://refractiveindex.info
